# Sandro Galea: the epidemiology of consequence

**DOI:** 10.2471/BLT.19.030719

**Published:** 2019-07-01

## Abstract

Sandro Galea talks to Gary Humphreys about the power of epidemiology and the need to change the way we talk about health.

Q: You started your career as a primary health care physician in northern Canada. How did you end up focusing on behavioural epidemiology?

A: It might seem counter-intuitive, but I wanted to have more of an impact.

Q: Can you explain that?

A: Well, I always wanted to make a difference. Even when I was in medical school, I was very interested in working in countries where the need for health-care professionals was acute. So, for example, during my training I spent four months in Papua New Guinea and a couple of months in Guatemala City at a time when there was considerable political upheaval there. After I graduated from medical school in Canada, I did my residency in the town of Geraldton in the north of the country. I was just one of three doctors in a town which was three hours from the nearest large urban area and a lot of my patients were from the First Nation population (indigenous peoples of Canada). These people were generally neglected and underserved by the health system. So, I knew I was making a difference, but I wanted to do more. I always had this desire to see what it was like to work in low-income settings and so I applied to work with Médécins sans Frontières in Somalia.

Q: How did that get you to focus on epidemiology?

A: In Somalia, I was the only doctor for about 350 000 people, so in many respects I felt like I was doing as much good clinically as I was ever going to be able to do, and yet it wasn't enough. I felt like I was pulling people out of a river, but I wasn't really understanding how they were falling into the river in the first place. And I couldn't help but feel that once I left, nothing was going to change. So, I started to ask myself some fairly fundamental questions. What is driving the burden of disease and death here? What is it, at the root, that makes people healthy? I realized that my medical school training had not really prepared me to answer those questions. I was trained in medicine at the University of Toronto, which at the time was a fairly traditional medical school. I knew nothing about public health. I knew nothing about prevention.

Q: You did your master’s of public health at Harvard University. Was that your first encounter with epidemiology?

A: Absolutely. I actually applied to do my master’s in Harvard’s health policy programme, but half way through the application process I realized that what I really wanted to do was specialize in epidemiology, and they were kind enough to accommodate me.

“There is great power in numbers. And when I say power, I mean power to effect change.”

Q: I understand that you went on to focus on the epidemiology of behaviour and mental health, in particular. Can you talk about that?

A: At Harvard, I became very interested in the intersection between social and psychiatric epidemiology. But once again I realized that I did not know enough and I decided to do a doctorate at Columbia University, which has a prestigious psychiatric epidemiology programme. I started at Columbia in 2000. So, in 2001, I was starting work on my doctoral thesis and my ideas were just forming when 9/11 happened – this massive traumatic event, a violent event, affecting large populations. As terrible as it was, it was an incredible opportunity to study the psychological impact of the event on people, up close and in real time. I ended up doing a lot more work on the psychological epidemiology of disasters and mass traumas. In fact, I spent a decade doing that kind of work, including work on mass shootings and the broader epidemiology of gun violence.

Q: You have argued in your articles and books for an ‘epidemiology of consequence’. What do you mean by that?

A: An epidemiology of consequence is an epidemiology that provides the evidence-base to inform public health action. Epidemiology should be at the heart of any public health thinking and action, and should address issues of consequence, such as gun control. And, yes, I have written extensively on that topic and have made a concerted effort to inject a sense of purpose into the field. Basically, my argument is for epidemiologists to quantify, to count what matters, what will contribute to creating a healthier world.

Q: You have suggested that epidemiologists should speak fearlessly, which suggests that speaking out is not always without consequences. Do you have personal experience of hostile reactions to the evidence you present?

A: In truth I'm largely protected from the harsh realities that many activists face. But I have run afoul of different groups at different times, including the National Rifle Association.

Q: Why was that?

A: They took exception to me challenging the prevailing national narrative on firearms.

Q: Challenging it in what way?

A: By presenting evidence regarding the commercial interests behind the narrative, and by presenting the reality of the gun violence epidemic itself – the fact, for example, that in the United States of America (USA) approximately 34 000 people have died from firearms annually since 2000, and two to three times that number are injured by guns. Despite this simple and appalling fact, no federal legislation has been enacted to reduce this threat to the populations’ health. I believe there is an important role for epidemiologists to point out the disconnect between emerging story lines and the underlying evidence, when this can be quantified. There is great power in numbers. And when I say power, I mean power to effect change. I have never seen epidemiology as a dry quantitative pursuit that is somehow annexed to health care, part of a monitoring function. I see it as a vital evidence-gathering tool that can be used as a basis for challenging the status quo. Epidemiology is also needed for policy formation, as well as the planning and the implementation of public health strategies. But it is more than that: epidemiology should be concerned with establishing the facts, the truth.

*Q: Most recently your focus has been on the health-care industry, notably in your book *Well: what we need to talk about when we talk about health*. Can you talk about the facts you are seeking to establish there?*

A: The facts I present relate to the central argument, which is that as a society we tend to conflate the concept of health with the practice of medicine, rather than seeing health as the product of broader determinants, such as poverty and social injustice. In other words, the forces that were affecting the people I was treating in Somalia. Needless to say, I draw on a lot of data, but some of the highlights include the fact that in the USA our health output per dollar is worse than that of any other high-income country. One of the reasons for this is that we are focusing on the wrong things. For about a decade we have been consumed by discussions around health coverage and in particular the Affordable Care Act, the challenges presented by its implementation and more recently, by the attempts by the current administration to “repeal and replace” it. While it is clear that the act was a long-needed step forward not least for the provision of health insurance coverage for 20 million Americans, who were previously without coverage, it probably will not have a huge impact on the country’s health indicators.

“Epidemiology should be concerned with establishing the facts, the truth.”

Q: So what should people be focusing on?

A: The resources that generate health such as nutritious food, education, a safe home in a safe neighbourhood and some level of economic security. Ensuring universal access to quality health care is also vital, of course.

Q: You are an academic and your other books are all written for specialists. You wrote this book for a general audience. Why?

A: I am trying to change the conversation around health. I’m drawing attention to the core forces that shape health, such as power, money, politics, place, love and hate, pain and pleasure, and choice. These forces determine whether we get sick or stay well, but they have tended to be neglected in the health conversation. In order to change the conversation, I felt it appropriate to address the widest audience possible.

Q: Is changing the conversation enough to bring about a change in health-care outcomes?

A: We will see. But I believe it can start a process that will lead to change. Changes in politics and policy tend to follow a change in the broader conversation. Institutions change in response to the broader conversation. And by the way, I consider optimism to be an important form of social activism.

Q: Do you have any indications that your message is being heard?

A: Most people I talk to say “Oh yes, I get it. I see the point you're making”. The challenge becomes how to move beyond platitudinous agreement to real engagement. But there is an appetite for change - of this I am certain. When I talk in the USA and I say to audiences how many of you are aware of the fact that there has been a downturn in life expectancy in this country for the past three years, and that this is the first time that we have had a downturn in life expectancy since the 1918 flu pandemic, perhaps 5% of people hold up their hands. Then, when I ask them “How many of you think that there should be the story on the front page of the papers every day?” everybody raises their hand. So there is this enormous mismatch between how much we care about health and how much we actually pay attention to what matters. That mismatch is frustrating, but I believe it also represents an opportunity. Because everybody thinks that we should be talking about why our health is nowhere near what it should be, and as long as everybody thinks that, the ground is ripe for a change in what we prioritize and how we approach bringing about change.

